# Efficient correction of a deleterious point mutation in primary horse fibroblasts with CRISPR-Cas9

**DOI:** 10.1038/s41598-020-62723-3

**Published:** 2020-05-04

**Authors:** Carlos Pinzon-Arteaga, Matthew D. Snyder, Cicera R. Lazzarotto, Nicolas F. Moreno, Rytis Juras, Terje Raudsepp, Michael C. Golding, Dickson D. Varner, Charles R. Long

**Affiliations:** 1 Department of Veterinary Physiology and Pharmacology, Texas A&M University, College Station, Texas USA; 2 University of Fortaleza, Fortaleza-CE, Brazil; 3 Department of Veterinary Integrative Biosciences, Texas A&M University, College Station, TX USA; 4 Department of Large Animal Clinical Sciences, Texas A&M University, College Station, TX USA; 5 Present Address: Department of Molecular Biology, University of Texas Southwestern Medical Center, Dallas, TX 75390 USA

**Keywords:** Genetic engineering, Animal biotechnology

## Abstract

Phenotypic selection during animal domestication has resulted in unwanted incorporation of deleterious mutations. In horses, the autosomal recessive condition known as Glycogen Branching Enzyme Deficiency (GBED) is the result of one of these deleterious mutations (102C > A), in the first exon of the *GBE1* gene (*GBE1*^102C>A^). With recent advances in genome editing, this type of genetic mutation can be precisely repaired. In this study, we used the RNA-guided nuclease CRISPR-Cas9 (clustered regularly-interspaced short palindromic repeats/CRISPR-associated protein 9) to correct the *GBE1*^102C>A^ mutation in a primary fibroblast cell line derived from a high genetic merit heterozygous stallion. To correct this mutation by homologous recombination (HR), we designed a series of single guide RNAs (sgRNAs) flanking the mutation and provided different single-stranded donor DNA templates. The distance between the Cas9-mediated double-stranded break (DSB) to the mutation site, rather than DSB efficiency, was the primary determinant for successful HR. This framework can be used for targeting other harmful diseases in animal populations.

## Introduction

For centuries humans have been performing phenotypic selection on plants, livestock and companion animals to suit their needs. Although this form of breeding has been successful in the establishment and improvement of many different breeds of plants and animals, we have unknowingly co-selected for deleterious mutations^1^. In horses, one of these deleterious mutations is the 102C > A nonsense mutation in the first exon of the *GBE1* gene, which causes the condition known as Glycogen Branching Enzyme Deficiency (GBED). This disease severely disrupts glycogen metabolism by decreasing the capacity of myophosphorylase to use glycogen as a substrate to liberate glucose^2^. This mutation is lethal in homozygotes, due to the lack of GBE activity in liver, cardiac and skeletal muscle^2–5^. An estimated 9% of Quarter Horse and Paint Horse lineages are heterozygotic carriers, which have half of the normal GBE activity^2–5^ but are otherwise asymptomatic. In addition, this disease is a significant cause of second and third-trimester abortion and foal mortality in the American Quarter Horse breed^3,6,7^.

Precise manipulation of eukaryotic genomes for the correction of monogenic diseases is now possible. This is due to the development of genome editing technologies based on programmable nucleases^8^, together with advances in both DNA sequencing and synthesis^9,10^. This advances can be applied to tackle the more than 6,000 identified genetic mutations linked to disease in humans and animals^11,12^.

Precise editing of a genome locus has historically been hampered by the overall low frequencies of homologous recombination, in which successful modification is achieved in one per a thousand transfected cells (0.001%)^13^. However, this changed in 1983 with the discovery that a site-specific double-stranded break (DSB) in genomic DNA stimulates a particular locus to exchange genetic information with either a homologous chromosome or an exogenously provided repair template^14^. Since this discovery, the field of genome editing has pursued the development of technologies to induce site-specific DSBs using engineered nucleases^15^: first with the zinc-finger nucleases (ZFNs)^8,16^, then transcription activator-like effector nucleases (TALENs)^17,18^, and more recently using the CRISPR-Cas system^19–21^.

DSBs are sensed by the cells endogenous DNA repair system^22–25^ which repairs the DSBs predominantly using the error-prone canonical non-homologous end joining (c-NHEJ) pathway. To a lesser extent, DSBs can be repaired by homologous recombination (HR), which is comprised of at least three sub-pathways: homology-directed repair (HDR), single-stranded annealing (SSA) and the recently recognized single-stranded template repair (SSTR)^26,27^.

The application of genome editing technologies can have a significant impact on animal health when combined with a unique and powerful tool that is not available in human medicine: reproductive cloning^28,29^. Producing cloned, gene-edited animals from modified cell lines has been widely reported^29–43^. For example, hornless cattle were recently produced by the introgression of the dominant P_C_ Celtic POLLED allele into primary cells of the Holstein breed using TALENs, which was followed by reproductive cloning^44,45^. However, genome editing to correct mutations in the equine genome has not been previously reported.

**Figure 1:**
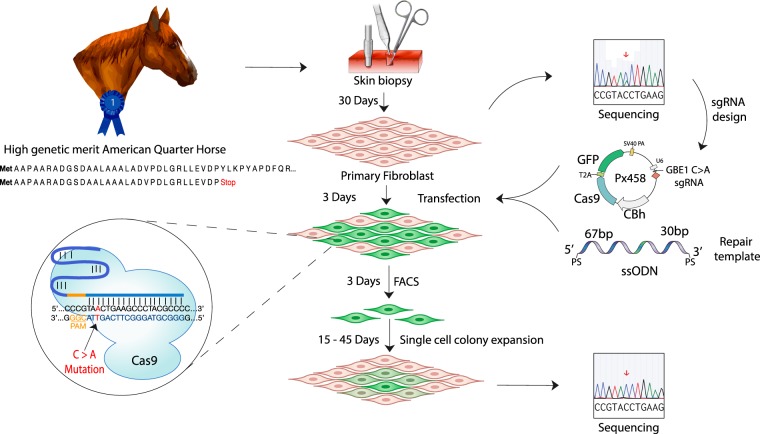
Experimental design for the correction of the GBE1^C>A^ mutation in primary equine fibroblasts. A skin biopsy from a GBE1^C>A^ heterozygous high genetic merit American Quarter Horse stallion was used to establish a primary fibroblast cell line. Different sgRNAs at varying distances from the mutation were designed and cloned into the CRISPR-Cas9 PX458 plasmid, which allowed for GFP selection via flow cytometry. Different single-stranded oligodeoxynucleotide (ssODN) repair templates were tested. Cells were co-transfected with the PX458 plasmid and the repair template. Cells were subsequently enriched by flow cytometry and single-cell colonies were recovered. The target region was sequenced for verification of the genetic correction.

Here for the first time, we utilized the CRISPR-Cas9 system to successfully correct, the GBED mutation in a heterozygous primary fibroblast cell line derived from an American Quarter Horse stallion. The long-term goal of our work is to use these corrected cell lines for somatic cell nuclear transfer (SCNT), to generate a cloned animal that maintains the genetic merit of its predecessor and is free of the GBED mutation (Fig. 1).

## Results

### Inhibiting apoptosis is beneficial for effective isolation of single-cell clones in primary horse fibroblasts

**Figure 2:**
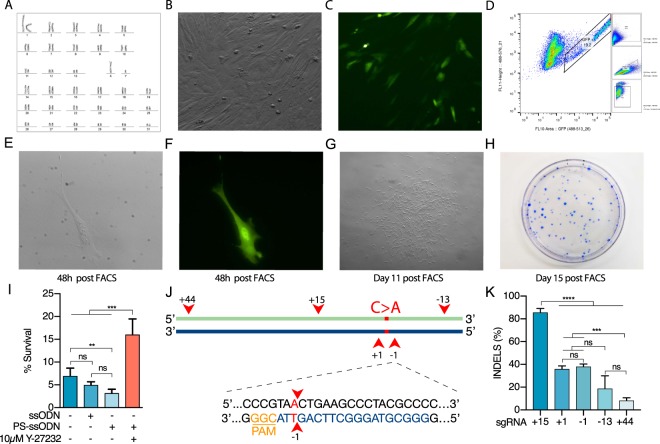
CRISPR-Cas9 Targeting of GBE1 locus in Primary Horse Fibroblasts. (**A**) Normal 64XY karyotype of the isolated equine primary fibroblasts. (**B**) Bright-field and (**C**) Fluorescent images 12 h after transfection. (**D**) Fluorescent activated cell sorting (FACS) enrichment of GFP+ cells. (**E**) Bright-field and (**F**) Fluorescent images of single-cells 48 h after sorting. (**G**) Single cell colony 11 days after sorting, (**H**) Coomassie blue stained colonies 15 days after sorting. (**I**) Effect of ROCK inhibitor Y27632 on cell viability post-sorting. (**J**) Schematic illustration of the different sgRNAs used to target the *GBE1*^102C>A^ mutation. The C to A mutation at base 102 is indicated in red, the red arrow indicates the location of Cas9-mediated DSB 3 bp upstream of the PAM of the designed guides (i,e. +44, +15, +1, −1, −13) (**K**) Insertions or deletions (INDELS) generated using the designed sgRNAs. Bars represent standard deviation (SD) among biological replicates. Kruskal-Wallis One-way ANOVA with Tukey’s HSD, adjusted value (p) p < 0.001 shown as **p < 0.001 shown as ***p < 0.0001 shown as ****.

In order to correct the mutation in a heterozygous American Quarter Horse stallion (Fig. 1), we first established a protocol for clonal isolation after transfection and enrichment by fluorescence-activated cell sorting (FACS). To this end, karyotypically normal primary fibroblasts derived from the heterozygous stallion (Fig. 2A**)** were transfected (Fig. 2B,C) and sorted by FACS for viable cells containing the construct (Fig. 2D). Sorted cells were plated at low density and after ~10–15 days of culture, single-cell colonies were recovered by using agarose embedded cloning rings^46^ (Fig. 2E–H). The initial viability of the sorted cells was low, as measured by the number of total colonies divided by the number of plated cells, with only 7.0 ± 1.7% of cells forming viable colonies.

To improve the post sorting viability, we added 10 µM of a Rho-associated protein kinase (ROCK) inhibitor (Y-27632) to our recovery medium. This compound blocks the ROCK-activated caspase signaling cascade leading to cellular apoptosis^47^ and has been reported to increase cellular proliferation and migration in human fibroblasts^48^ (Fig. 2I). This compound improved the post-sorting survival, for a total average viability post FACS of 16.0% ± 3.4 (p < 0.0001, Kruskal-Wallis One-way ANOVA with Tukey’s HSD F(3,15) = 46.8) (Fig. 2I).

### Cas9-induced high DSB efficiency does not imply high HR efficiency

Once we established a protocol for the isolation of single-cell clones; we designed five different sgRNAs flanking the *GBE1*^102C>A^ mutation at variable distances (Fig. 2J, Supplementary Table 1), taking computationally predicted off-target activity and folding efficiency into consideration. These sgRNAs were named according to the Cas9-induced DSB relative to the C > A mutation (e.g sgRNA -1 cutting 1 bp 3′ of the mutation, as Cas9-induced DSB occurs 3 bp upstream of the protospacer adjacent motif (PAM), Fig. 2J). From the different sgRNAs tested, sgRNA +15 displayed the greatest INDEL efficiency (p < 0.0001, Kruskal-Wallis One-way ANOVA with Tukey’s HSD, F(4,10 = 84.44)), while the other sgRNAs (+44, −13, +1, and −1) displayed comparable levels of activity except for sgRNA+44 (Fig. 2K).

After successfully targeting the *GBE1* locus, we then pursued the correction of the C > A mutation by homologous recombination using single-stranded oligodeoxynucleotides (ssODN) as repair templates. We observed variable INDEL formation (8.3–85.7% n = 124) but no HR, when distal sgRNAs (+44, +15, −13) were co-transfected with and without a ssODN repair template with a 100 bp 5′ homology arm and a 30 bp 3′ homology arm from the mutation ((100–30) ssODN, Supplementary Fig. 1, Table 1).

**Table 1:** Distal sgRNAs with and without a (100-30) ssODN.

sgRNA	+(100- 30) ssODN RT			−(100- 30) ssODN RT		
	n Sequenced	% INDELS	% HR	n Sequenced	% INDELS	% HR
+44	16	18.8^a^	0.0	12	8.3^a^	0.0
+15	33	81.8^b^	0.0	28	85.7^b^	0.0
−13	19	15.8^a^	0.0	16	18.8^a^	0.0

Table comparing the results of distal sgRNAs with (+ssODN RT) and without (+ssODN RT) a 100-30 single-stranded oligonucleotide (ssODN). The sgRNA describes the location of the cutting site with respect of the mutation location (0), + indicates upstream or 5′ and - indicates downstream or 3′. The percent of INDELS was calculated as the number of sequenced colonies that showed insertions or deletions in the chromatograph analysis divided by the total number of sequenced colonies. The percent of HR was calculated as the number of sequenced colonies that did not presented a double peak in the mutation site and did not had any observable alteration in the chromatograph analysis divided by the total number of sequenced colonies. Values with different subscripts are different by chi-square test (P < 0.05).

Due to the high level of INDEL formation with sgRNA+15, we hypothesized that HR rates could be improved using this sgRNA together with small molecules previously reported to enhance HR^49^. To test this we used the RAD-51 stimulatory compound RS-1 (15 µM)^50^ and the DNA-Ligase IV inhibitory compound SCR7 (80 µM)^51^ alone or in combination with the previously used 100-30 ssODN. However, under these conditions, no HR events were obtained and similar INDEL rates were observed between groups (Chi-square test P > 0.05, Table 2).

**Table 2:** RS-1 and SCR7 effect on sgRNA +15 with a (100-30) ssODN.

Treatment	n Sequenced	% INDELS	% HR
No Compound	33	81.8	0.0
15 µM RS-1	36	86.1	0.0
80 µM SCR7	43	79.1	0.0
Combined	43	81.0	0.0

Comparisons of the RAD-51 stimulatory compound RS-1 and the DNA-Ligase IV inhibitory compound SCR7 or their combination when using sgRNA+15 a ssODN. The percent of INDELS was calculated as the number of sequenced colonies that showed insertions or deletions in the chromatograph analysis divided by the total number of sequenced colonies. No HR positive colonies were obtained. No differences were observed between treatment groups using a chi-square test (P < 0.05).

We then hypothesized that our sgRNA +15 could also be cutting the newly corrected allele, so we decided to incorporate a silencing mutation in the sgRNA +15 PAM together with a longer 5′ arm (168 bp), as more extensive homology arms are normally associated with better HR rates. We again tested this new repair template in combination with RS-1 and SCR7, but no HR events were obtained (Supplementary Table 2, Supplementary Fig. 1).

The observation that the sgRNA with the highest INDEL efficiency (sgRNA +15) did not produced detectable HR rates, even in the presence of small molecules or a silenced PAM, led us to hypothesize that cleavage efficiency was not the main factor driving HR and we proceeded to evaluate DSB proximity and repair template stability as possible factors for successful repair by HR.

### Proximity of Cas9-induced DSB to the mutation and repair template stability are major determinants of HR efficiency

In order to improve the stability of the repair template we incorporated phosphorothioate (PS) modifications, which have been reported to increase HR rates by protecting repair templates from exonuclease activity^52^. Additionally, Liang, *et al*.^53^ reported that a small asymmetric repair template was very efficient in HR, so we decided to shorten our 5′ homology arm ((67-30) PS-ssODN). We noticed that when a PS modified ssODN was used the viability was decreased (3.2% ± 0.9 (p < 0.001)) without the use of the Y-27632 compound, as compared to control cells and a non-modified ssODN (Fig. 2I). This indicates that extending the half-life of the repair template can have toxic effects in primary cells and the use of apoptosis inhibitory compounds is warranted, as the addition of Y-27632 produced an approximately 8-fold increase in the number of viable colonies (Fig. 2I).

**Figure 3:**
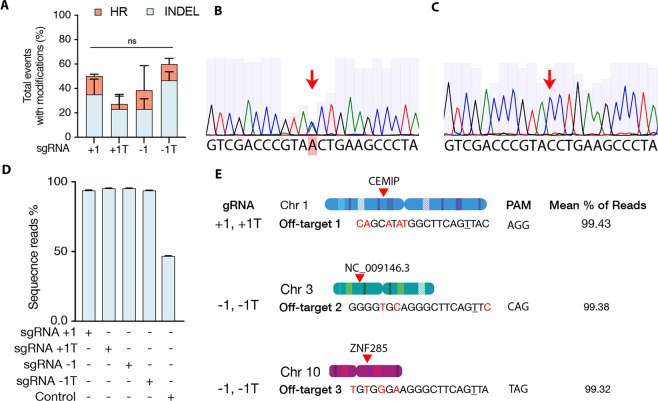
Gene editing outcomes of proximal sgRNAs. (**A**) Total events with modifications showing the percent of INDELS and HR of proximal sgRNAs (+1, −1), and their truncated versions (+1T, −1T) with an asymmetric (67-30) PS-ssODN. Chi-square test (P > 0.05). (**B**) Sanger sequencing chromatograms of control unmodified colony: red arrow indicates the double peak showing the C (blue) to A (green) conversion in one of the alleles, representing the heterozygous state of the horse. (**C**) Representative chromatogram of gene-edited single-cell clone. Note there is an absence of the double peak observed in the control colony and the height of the both adjacent cytosines read peaks is approximately the same, indicating the correction of the mutation in the mutated allele. (**D**) Next generation sequencing (NGS) results of a subset of corrected clones and control mapping to the wildtype allele (Error bars represent SD among sequencing probes). (**E**) Genomic locations of the three off-target loci for guides +1, −1 and their truncated versions analyzed by NGS. Coding regions were prioritized.

When we tested our hypothesis by co-transfecting the proximal sgRNAs (+1, −1) together with the (67-30) PS-ssODN we observed 15.0% HR (n = 20) for sgRNA +1 and 15.4% (n = 13) HR for sgRNA −1. To decrease the probability of computationally predicted off-target effects^54^ (Supplementary Dataset 2), truncated versions of these sgRNAs (+1T, −1T) were used, which yielded 4.3% (n = 23) and 12.5% (n = 16) HR, respectively (Fig. 3A–C, Table 3).

**Table 3:** Proximal sgRNAs with and without a (67-30) PS-ssODN.

sgRNA	+(67-30) PS-ssODN			−(67-30) PS-ssODN		
	n Sequenced	% INDELS	% HR	n Sequenced	% INDELS	% HR
+1	20	35.0	15.0	21	33.3	0.0
+1T	23	30.4	4.3	7	57.1	0.0
−1	13	23.1	15.4	8	37.5	0.0
−1T	16	46.6	12.5	12	41.7	0.0

Comparisons of INDELS and HR frequencies between cells treated with proximal sgRNAs with and without an asymmetric (67-30) PS-ssODN. The sgRNA describes the location of the cutting site with respect of the mutation location (0), + indicates upstream or 5′ and − indicates downstream or 3′. Truncated (17 nt) sgRNAs are represented by a (T). The percent of INDELS was calculated as the number of sequenced colonies that showed insertions or deletions in the chromatograph analysis divided by the total number of sequenced colonies. The percent of HR was calculated as the number of sequenced colonies that did not presents a double peak in the mutation site and did not exhibit any observable alteration in the chromatograph analysis divided by the total number of sequenced colonies. No differences were observed between treatment groups using a chi-square test (P = 0.28).

DNA from control and gene-edited clones was sent for independent analysis by the Texas A&M Genetics Laboratory for carrier status and type from 13 microsatellite markers specific to *Equus caballus*. Colonies analyzed are free from carrier status and from the same individual as the non-transfected control which is carrier of the GBED allele.

In order to verify that large deletions or mutations had not caused a failure to amplify the PCR product, genomic DNA from control and a subset of edited colonies were submitted for independent verification by targeted next generation sequencing (NGS) at the Genome Engineering and iPSC Center of the University of Washington School of Medicine. NGS revealed that three clones originally identified by our PCR product sequencing and TaqMan Assay as correctly repaired indeed contained detectable deletions. The remaining sequenced clones show more than >90% (93.9–98%) of reads aligned to the wildtype sequence with control showing only 50% with the remaining 50% corresponding to the mutated allele (Fig. 3D). Finally, we also sequenced the three most common predicted off-target sites among all guides and no INDELS were detected (Fig. 3E).

Taken together, we have created a framework for efficient genome editing in primary equine fibroblast and demonstrate that proximity of the cut site to the mutation site and stability of the repair template are the main factors affecting HR in our experiments.

## Discussion

Traditional phenotypic selection and breeding in companion and agricultural animals has unknowingly co-selected and propagated deleterious mutations in animal populations. Monogenic diseases are surprisingly prevalent across many equine breeds, including conditions such as the Hyperkalemic Periodic Paralysis (HYPP), Severe Combined Immunodeficiency (SCID), Overo Lethal White Syndrome (OLWS), Junctional Epidermolysis Bullosa (JEB), Malignant Hyperthermia (MH), Hereditary Equine Regional Dermal Asthenia (HERDA), type 1 Polysaccharide Storage Myopathy (type 1 PSSM) and the Glycogen Branching Enzyme Deficiency (GBED) (Online Mendelian Inheritance in Animals, OMIA, http://omia.org/). With the discovery of CRISPR-Cas9 and advances in DNA sequencing and synthesis^10,55^, we now have the genetic tools to detect, correct and eliminate these diseases from animal populations. The main objective of this study was to correct the GBED mutation in a heterozygous American Quarter Horse stallion cell line using CRISPR-Cas9. The optimized conditions presented in this work could be used to obtain high HR-based repair rates in primary cells for the future targeting of other genetic diseases.

Many genetic engineering strategies incorporate DSBs in close proximity to the target region to be repaired^56–58^. In primary equine fibroblasts, under the conditions of this study, only the sgRNAs targeting the mutation site in very close proximity (+1, −1) were capable of producing HR events (Table 3). Our results agree with other published reports and identify proximity as one of the most critical factors for HR^52^. The inability to detect HR events with sgRNAs guiding Cas9-DSB distal (>~10 bp) from the mutation, could be due to our small sampling size or the low DSB efficiency (i.e sgRNA −13). We did not observe any HR events when we used a sgRNA that induced Cas9-mediated INDELs with high efficiency (sgRNA +15) despite many different conditions. (Tables 1 and 2, Supplementary Table 2). Another factor that can influence HR rates is allele specificity, as Cas9 does not tolerate PAM-proximal mismatches^59^, proximal sgRNAs (+1, −1) could only target the mutated allele and distal sgRNAs(+44, +15, −13) could target both alleles.

The use of the RAD-51 stimulatory compound RS-1 (15 µM)^60^ and the DNA-Ligase IV inhibitory compound SCR7 (80 µM)^61^, at the concentrations tested, did not yield any benefit in improving HR rates (Table 2, Supplementary Table 2). It is important to note that the concentration of SCR7 in the original publication of Chu, *et al*., was modified to 1 µM after our experiments were performed^62^, however this molecule has been highly controversial and other reports indicate a very high variability of this molecule between different experimental systems^63^ or that SCR7 is neither a selective nor a potent inhibitor of the human DNA ligase IV^64^. It is also unclear if this molecule can effectively inhibit equine DNA ligase IV, although it shares a 91% sequence similarity with the human homolog. RS-1 is a Rad51 stimulatory molecule identified by a small molecule screen where it was shown to stabilize the association of RAD51 with DNA^60^. Rad51 is an essential player in the HDR pathway that displaces the replication protein A (RPA) to form a RAD51–ssDNA nucleofilament recombinase, which is required for homology search, strand invasion and ultimately the repair of the DSB lesion^65^. Song *et al*.^50^, reported RS-1 improves HR rates 2–5 fold at a concentration of 7.5 µM *in vitro* and *in vivo* (from 4.4 to 26.1%). RS-1 was also shown to improve HR rates in HEK293 cells (from 3.5 to 21%) and U2OS cells (from 1.9 to 2.4%) at a concentration of 10 µM, but this varied depending on the loci and transfection methods^66^. It is important to note that a HR repair with a ssODN has been recently referred to as single-stranded template repair (SSTR)^26,67^ and it has been identified as a RAD51/BRCA2 independent pathway^68^, so the type of repair template used is one of the possible explanations as why we did not observe any benefit of the RS-1 molecule.

Hollywood *et al*.^69^, reported that after analyzing CRISPR-Cas9 repair tracts in the human CFTR gene, they found 90% of the editing events in the CFTR locus were long continuous repair tracts in excess of 100 bp from the DSB with no bias towards bi-directional or uni-directional correction. Based on this finding, they created sgRNAs that induced a Cas9 DSB 100 bp away from the target and obtained 1.9% HR events when providing a repair plasmid that harbored seven nucleotide differences with ~2 kb homology arms. They suggested that there is a ~200 bp window in which to select gRNAs for template-dependent editing. We could not employ long flanking homology arms due to the lack of the 3′ intronic sequence missing in the EquCab2.0 assembly at the initial time of this study and that we were unable to sequence despite numerous efforts (data not shown).Furthermore, the PCR product used to amplify the targeting region was too short to preform bulk gRNA cutting assessment with standard assays such as T7 endonuclease assay.

The lack of sequence information and the GC content flanking the GBED mutation limited our PCR product size resulting in a failure to detect large deletions. Using the new EquCab3.0 assembly, we performed targeted NGS on a subset of edited clones. We were unable to extend the coverage of the NGS reads for more than 70 bp 3′ of the GBED mutation due to the high CG content. Sequenced clonal cell lines show more than >90% (93.9–98%) of reads aligned to the wildtype GBED free sequence, whereas the heterozygous control DNA from the affected horse shows only 50% with the remaining 50% corresponding to the mutated allele. NGS revealed that three clones originally identified by our PCR product and TaqMan Assay as correctly repaired indeed had deletions that we had originally missed (Supplementary Dataset 1). Analysis of the NGS reads using both the SNP_test and raw_wt_test by CRIS.py^70^ confirmed that large deletions and/or mutations were not present in the genetically corrected cell lines. Finally, we performed targeted NGS for three predicted off-target sites among guides +1, −1 and their truncated versions. The sites were selected by prioritizing coding regions among possible off-target loci. No INDELS were detected in the NGS data among these sites in all the different single cell colonies sequenced. This illustrates the importance of careful analysis of the target site, as well as a reliably sequenced and annotated genome^10^ for the successful gene editing of agricultural and companion animals.

A single-stranded donor of approximately 90 nt in length was reported to produce high HR rates by Yang, *et al*., in human stem cells^57^. This finding was later confirmed by Liang, *et al*.^52^, who reported that an asymmetric ssODN (67-30) with PS modifications achieved up to 56% HR in HEK293 cells. A mechanistic explanation was proposed by Richardson, *et al*.^71^, who suggests that after the interaction of Cas9 with target DNA, Cas9 releases the PAM-distal non-target strand after cleavage but before complete dissociation. Based on this finding, Richardson, *et al*., rationally design an asymmetric ssDNA that matches the displaced strand, which increased HR rates by up to 60% in the absence of any chemical intervention^71^. In our work, we were able to obtain HR rates (up to 20%) when using an asymmetric ssODN with PS modifications ((67-30) PS-ssODN) together with proximal sgRNAs that induced a DSB within one nucleotide of the mutation site.

Editing of therapeutically relevant primary cells and tissues remains challenging. Exogenous DNA in primary cells stimulates the cytosolic DNA sensor cGMP-AMP (cGAMP) synthase (cGAS), which uses the second messenger cGAMP to activate the stimulator of interferon (INF) genes (STING). This leads to the production of Type I interferon and pro-inflammatory cytokines in an INF regulatory factor 3 (IFR3) and nuclear factor κβ (NF-κβ) dependent manner^72–74^. One recent improvement has been the delivery of Cas9 protein together with *in vitro* transcribed sgRNAs as a ribonucleoprotein complex (RNP), which bypasses these cytosolic DNA sensors and is therefore better tolerated in primary cells. Additionally Cas9 RNP delivery reduces the half-life of the complex and therefore decreases off-target effects^75^. However, Cas9 RNP triggers RNA-mediated innate immune responses, that can be avoided by a phosphatase treatment of sgRNAs to remove the 5′-triphosphate^76,77^. In this work we used CRISPR-Cas9 as a plasmid-based system because of the ability to enrich for transfected cells, which could explain the low viability post sorting we observed. New methodologies to fluorescently label the Cas9-RNP have been developed and these should be preferable in future work^78^.

To improve viability after transfection, we added 10 µM of the Rho-associated protein kinase (ROCK) inhibitor (Y27632) to our FACS recovery media, since this compound blocks the ROCK-activated caspase signaling cascade leading to cellular apoptosis^47^ and has been reported to increase cellular proliferation and migration in human fibroblasts^48^. We observed an approximately 6-fold increase in the number of viable colonies, for an average post FACS viability of 16.0 ± 0.9% when PS-ssODN were used (Fig. 2I). These results agree with other reports that inhibiting apoptosis is crucial for recovering edited colonies in primary cells, specifically the combined use of a p53 inhibitor (PFTα) and basic fibroblast growth factor (bFGF) in primary porcine cells^79^.

Besides the proximity and type of repair template, another possibility for the lack of HR with the distal sgRNAs at the GBE1 locus is the genomic context and the chromatin state at this locus. It has been shown that Cas9 diffusion and chromatin binding is reduced but not eliminated at heterochromatic regions^80^. Nucleosomes can directly impede Cas9 binding and cleavage, while chromatin remodeling can restore Cas9 access^81^. Miyaoka *et al*., showed that depending on the genomic context, more HR than NHEJ can be induced^82^, thus, the repair of the DSB is also influenced by the chromatin context. Although the repair of heterochromatic DSBs is just starting to be understood, it is known that pericentrometric heterochromatin relies on the relocalization of repair sites to the nuclear periphery before Rad51 recruitment and repair progression^83,84^. Due to the tissue-specific expression of the GBE1 gene, it is likely that in primary fibroblasts this gene is silenced and the DSB repair mechanisms might be influenced by the chromatin state.

C > A mutations can occur at relatively high rates due to spontaneous cytosine deamination^85^. Base editors provide a unique advantage to the correction of pathogenic mutations as there is no requirement for the introduction of a DSB or the HR machinery. Adenine base editors have been developed that can mediate the conversion of A•T to G•C in genomic DNA^85,86^, but the A•T to C•G (wild type sequence) or A•T to T•A (silence mutation) conversion required to correct the GBED mutation is not possible to date.

Although differences in HR or INDELS among proximal sgRNAs did not show statistically significant differences due to the small sample size (Fig. 3A, Table 3), we observed that truncating sgRNA+1 tended to increase INDEL rates, likely to the elimination of four continuous Gs at the 5′ end of the guide, as these are known to form a guanine-tetrad secondary structure that can decrease guide efficiency^87^ (Supplementary Table 1). We did not observe any off-target effects in the three evaluated loci with either the full length or truncated guides.

Producing cloned, gene-edited animals from modified cell lines has been widely reported^29–43^. Recent reports on bioinformatic analysis^88,89^ of the offspring of the gene-edited hornless bull^44,45^, homozygous for the dominant P_C_ Celtic POLLED allele, revealed that there were unintentional integrations of the donor plasmid used as a repair template^88,89^. In this work we used ssODNs as repair templates that could bypass this type of unintended edits, but the risk of unintended integrations of the Cas9 plasmid could exist. The framework created in this study can be applied for the future generation of gene-edited colonies using Cas9-RNP that be then used for reproductive cloning.

In conclusion, we were able to correct the GBED mutation with moderate efficiency (~20%) when using Cas9 and proximal sgRNAs creating DSB within one nucleotide of the intended target site. We have also shown that sgRNA INDEL efficiency does not always correlate with the incorporation of HR events, even in the presence of small molecule compounds that have been reported to improve HR.

## Materials and Methods

All methods were performed in accordance with Texas A&M University guidelines, biological safety and institutional animal care and use committee.

### Establishing primary fibroblast cell line and culture

A skin biopsy from an American Quarter Horse Stallion was donated to our laboratory by the horse owner who collected it an aseptic manner and under local anesthesia. All work was done under the supervision of a licensed veterinarian using protocols approved by the Texas A&M University IACUC. The tissue sample was placed in 1x Ca^2+^ and Mg^2+^ free DPBS (14040133, Life Technologies^®^) and transported on ice. The tissue was washed in a 0.2% (v/v) chlorhexidine gluconate in DBPS followed by two DPBS washes. Tissue was trimmed into small pieces (<5 mm) and washed through a series of 1xDPBS washes in a 15 ml conical tube; where the sample was mixed by inversion, and the tissue was allowed to briefly settle to the bottom and supernatant was aspirated. The tissue was then suspended in Dulbecco’s Modified Eagle Medium with nutrient mixture F-12 (DMEM/F12, 11320082, Life Technologies^®^) supplemented with 10% fetal bovine serum (Premium Select FBS, S11595, Atlanta Biologicals), 1x antibiotic-antimycotic (Anti-Anti^™^, 15240062, Thermo^®^) and 50 µg/ml gentamicin (15750060, Life Technologies^®^). The tissue sample was finally placed in a T25 tissue Falcon tissue culture flasks (353014, Corning^®^) and cultured at 37 °C in a 5% CO_2_ and 5% O_2_ humidified incubator (Nuaire) to reduce oxidative stress. Cells were passaged when 80% confluence was reached, in a split ratio of no more than 1 to 3.

### Karyotyping

A normal karyotype integrity is essential for somatic cell nuclear transfer (SCNT) procedures^90^. For this, primary cells were grown for chromosome preparations following standard procedures as described by Raudsepp *et al*.^91^, and karyotyped according to GTG-banding width^92^.

### Single guide RNA design

sgRNAs were designed using different bioinformatics web-based tools followed by a manual sequence verification. The different programs used were the Broad institute CRISPRko which uses the on-target scoring described by Doench, *et al*.^93^, and the WU-CRISPR tool of the Washington University described by Wong, *et al*.^87^, that evaluates the gRNA folding. All DNA sequences were manipulated using Benchling^®^^94^ and ApE (A plasmid editor, http://jorgensen.biology.utah.edu/wayned/ape/). RNA folding was assessed using the Mfold 2web server for nucleic acid folding and hybridization prediction. Each guide and its complementary sequence were ordered as synthetic 25 nm oligos from Thermo^®^ with attached BbsI cloning sites: Sense: 5′–CACCGNNNNNNNNNNNNNNNNNNN–3′ and antisense: 3′–CNNNNNNNNNNNNNNNNNNNCAAA–5′ (Supplementary Table 1).

### Single guide RNA cloning into the PX458 plasmid

The designed guides were cloned into the PX458 (pSpCas9(BB)-2A-GFP) plasmid (Addgene, #48138). Synthetic oligonucleotides were suspended in water to 100 µM and then 10 µM of oligos where phosphorylated using T4 PNK (M0201S, NEB) at 37 °C for 1 hour. A total 0.5 µM mix of both oligos, was denatured at 95 °C for 5 minutes and annealed by slowly cooling down at room temperature. The PX458 plasmid was digested with FastDigest BbsI (ER1011, Thermo^®^) for 1 hour at 37 °C, dephosphorylation was carried out with alkaline phosphatase (rSAP, M0371S NEB) for 30 minutes and the plasmid was run on a 1% agarose gel and subsequently purified using the QIAquick Gel Extraction Kit (28704, Qiagen^®^). We ligated 50 ng of the linearized vector and 1 µl of the annealed oligos (0.5 µM) using Quick ligase^™^ (11635379001, Roche^®^) for 1 h at room temperature. One Shot TOP10 competent *E. coli* cells (C404003, Thermo^®^) were transformed following manufacturer instructions and plated in 100 µg/ml Ampicillin (A5354, Sigma) LB agar plates followed by an overnight incubation at 37 °C. Insert was verified by colony PCR using the sense oligo as a forward primer and a common CBh reverse primer: 5′ GTCAATAGGGGGCGTACTTGG 3′, at a 50 °C annealing using the HiFi PCR premix (Clonetech^®^). A Maxiprep (Qiagen^®^) was performed followed by a final verification by Sanger sequencing using the LKO forward primer for the human U6 promoter: 5′ GACTATCATATGCTTACCGT 3′.

### Design of repair templates

All repair templates were centered to the GBED mutation site (GenBank: AY505110.1), with a 30 bp 3′ homology arm and a variable length 5′ homology arm. All single-stranded oligodeoxinucleotide (ssODN) repair templates were ordered as PAGE Ultramer^®^ DNA Oligos from IDT^®^ and reconstituted to 100 µM concentration in nuclease-free water. All DNA sequences were manipulated using Benchling. (Supplementary Material 1, Supplementary Fig. 1).

### Transfection of equine fibroblasts

In order to improve attachment and viability after transfection, plates were pre-coated with 0.1% gelatin in 1xDPBS and incubated at 37 °C for 1 h. Early passage cells were passed as described above and plated at density of 3 × 10^5^ cells per well in a 6 well plate. Cells were transfected at 80% confluency using Lipofectamine 3000^™^ (L3000001, Thermo^®^). For this, each well was transfected with 1 µg of CRISPR-Cas9 plasmid (PX458 with sgRNA) and 3 µl of the 10 mM ssODN (∼600 ng/µl). Media was replaced 12 h after transfection.

### Flow cytometry enrichment of transfected. Cells

After verifying GFP expression under an inverted fluorescence microscope (Nikon Eclipse TE300), cells were trypsinized, suspended in sorting media (10% FBS in DPBS, 1xAnti-Anti and 1 mM EDTA) and transported on ice to the Texas A&M CVM flow cytometry facility. Before the flow sort, cells were stained with 2.5 ng/ml of propidium iodide (PI). PI negative and GFP positive cells were sorted into 45% FBS, 45% DMEMF12, 10% conditioned media from healthy cells, 1x Anti- Anti and 10 µM Y-27632, ROCK inhibitor (Stemcell^®^). Cells were plated at a density of 1000 cells per 150 mm dish for subsequent single-cell colony isolation.

### Single-cell colony isolation

After ~10 days of culture single-cell colonies were recovered using agarose-embedded cloning rings for single-cell clone isolation as described by Mathupala, *et al*.^46^. Briefly, single cells colonies were marked (MBW10020, Nikon) and cloning cylinders carefully placed around clones with sterile curved forceps; then one percent (w/v) LMP agarose (A9414, Sigma^®^) in 1xDBPS (37 °C) was then slowly dispensed dropwise around the outside of the cloning cylinders. Cells were lifted with 40 µl of 0.05% trypsin-EDTA (Invitrogen^®^) at 37 °C for 5 minutes and then 100 µl of culture media was added to inactivate the trypsin and pipetted several times to detach and suspend cells before moving each colony to a 48 well plate. Subsequent passages to larger wells were made as the cells reached ~80% confluency. Cells from 6 well plates were passaged and half were cryopreserved in 10% DMSO, 45% FBS, 45% DMEM-F12 media using an isopropyl alcohol freezing container (5100-0001, Thermo^®^). The other half was used for DNA extraction.

### Colony genotyping

DNA was extracted using the DNeasy Blood & Tissue Kit (Qiagen^®^) with the addition of 1 µl of tRNA (10 µg/µl) for improved DNA yield. DNA was eluted in nuclease-free water and the concentration was measured using the nanoDrop^™^ spectrophotometer (Thermo^®^). PCR was performed with approximately 200 ng of genomic DNA by using 2.5 units of the the HotStarTaq plus DNA polymerase (Qiagen^®^) with 1x Q solution, 100 µM dNTp Mix, and 0.1 µM of the forward and reverse primers in a 50 µl reaction. The PCR was performed at an annealing temperature of 57 °C with an extension time of 30 s for 40 cycles. PCR products were purified using the QIAquick^™^ PCR purification kit (Qiagen^®^) and eluted with nuclease-free water. Sanger sequencing was performed at the Texas A&M Institute for plant Genomics and Biotechnology. Forward primer: 5′ CTCGCCGCTATAAAGGGCCCC 3′, reverse primer: 5′ TGCGCTGGAAGTCCGGGG 3′. All chromatogram files were aligned in Benchling^®^. INDELs edits were analyzed using CRISPR-ID^95^ and ICE (Inference of CRISPR Edits)^96^.

### Microsatellite analysis and RT-PCR taqman assay

Genomic DNA from gene edited, non-carrier single-cell colonies identified by Sanger sequencing and control sample, were sent to the Texas A&M Genetics Laboratory for independent TaqMan DNA typing and carrier status verification. A total of 13 microsatellite markers (AHT4, AHT5, ASB17, ASB23, HMS6, HMS7, HTG4, VHL20, HMS3, ASB2, HTG10, LEX33 and HTG6) specific to Equus caballus were used^97,98^. All markers are included in the panel were recommended by the International Society for Animal Genetics. GBED carrier status was determined by RT-qPCR TaqMan Assay. Forward primer: CCTGGGCCGCCTTCT. Reverse primer: GCGCTGGAAGTCCGGG. VIC probe: CCCGTACCTGAAGCC. FAM probe: CCCGTAACTGAAGCC.

### Targeted next generation sequencing

DNA from control and a subset of edited colonies were sent for independent targeted next generation sequencing (NGS) at the Genome Engineering and iPSC Center of the University of Washington School of Medicine. Analysis was performed using CRIS.py^99^. The sequence of the guide RNAs +1, −1 and their truncated versions (Supplementary Table 1) were used to match the NGS output at the GBED loci. Additionally, 3 off-target loci were identified using Benchling Off-target finder and submitted for NGS and output matched to the sequence in EquCab3.0 assembly. PCR and sequencing errors such as C homopolymers were subtracted from sequencing reads before final analysis (Fig. 3D,E. Supplementary Dataset 1). Quality control measures (SNP_test and raw_wt_test ratios) were determined for each sequenced cell line to detect the potential for mutations or large deletions.

### Statistical analysis

All experiments were performed using three or more independent biological replicates unless otherwise indicated. Statistical analyses of categorical variables were conducted using Pearson’s Chi-square or Fisher’s exact test^99–101^. For viability and fluorescence intensity analyses, after verifying for the assumptions of equal variance and normality, P values were calculated using One-Way ANOVA with Tukey’s HSD^102^. Unless otherwise indicated, error bars represent standard deviation. Analyses were performed with Prism^™^ (Graphpad^®^). Values with different subscripts are different (P < 0.05).

## Supplementary information

Supplementary information.

Supplementary Dataset 1.

Supplementary Dataset 2.
