# Supplementary material for: Efficient correction of a deleterious point mutation in primary horse fibroblasts with CRISPR-Cas9
Source: Sci Rep. 2020 May 4;10:7411. doi: 10.1038/s41598-020-62723-3 (PMC7198616; doi:10.1038/s41598-020-62723-3)
Supplement: Supplementary file 1 — Supplementary information. [file 41598_2020_62723_MOESM1_ESM.pdf]

Efficient correction of a deleterious point mutation in primary horse fibroblasts with CRISPR-Cas9

Carlos Pinzon-Arteaga[1,ⱡ], Mathew Snyder[1], Cicera R. Lazzarotto[2], Nicolas Moreno[1], Rytis Juras[3], Terje Raudsepp[3], Michael C. Golding[1], Dickson Varner[4] and Charles R. Long[1, *]

1. Department of Veterinary Physiology and Pharmacology, Texas A&M University, College Station, Texas, USA.
2. University of Fortaleza, Fortaleza-CE, Brazil.
3. Department of Veterinary Integrative Biosciences, Texas A&M University, College Station, TX, USA.
4. Department of Large Animal Clinical Sciences, Texas A&M University, College Station, TX, USA.
ⱡPresent address: Department of Molecular Biology, University of Texas Southwestern Medical Center, Dallas, TX 75390, USA

*Corresponding author: clong@cvm.tamu.edu

Supplementary Information

Supplementary Material 1.

(100-30) ssODN repair template

GCTCCGACGCGGCGCTGGCGGCGGCCCTGGCGGACGTGCCCGACCTGGGCCGCCTTCTGGAGGTCGACCCGTAC
CTGAAGCCCTACGCCCCGGACTTCCAGCGCA

(168-30) ssODN repair template with PAM silence mutation

GGCCACTCCGCGGAGCTCGTTCCCGCTCGAGCGGCTCGGGCCTCGGCTACTCGGGCTGCGGCCGAAGATGGCGG
CGCCGGCGGCTCGGGCCGACGGCTCCGACGCGGCGCTGGCGGCGGCCCTGGCGGACGTGCCCGACCTGGGCCG
GCTTCTGGAGGTCGACCCGTACCTGAAGCCCTACGCCCCGGACTTCCAGCGCA

(67-30) ssODN GBED PS asymmetric repair template

A*C*G CGG CGC TGG CGG CGG CCC TGG CGG ACG TGC CCG ACC TGG GCC GCC TTC TGG AGG TCG ACC
CGT ACC TGA AGC CCT ACG CCC CGG ACT TCC AGC* G*C

GBE1 5’ UTR Exon 1 CDS.

CTCGCCGCTATAAAGGGCCCCGGGCCGCAGCCGCTCGCCTCGGCGTCCTCGGCTCCGCCCTCGCGCCGGCCACTCC
GCGGAGCTCGTTCCCGCTCGAGCGGCTCGGGCCTCGGCTACTCGGGCTGCGGCCGAAG**ATG**GCGGCGCCGGCGG
CTCGGGCCGACGGCTCCGACGCGGCGCTGGCGGCGGCCCTGGCGGACGTGCCCGACCTGGGCCGCCTTCTGGAG
GTCGACCCGTACCTGAAGCCCTACGCCCCGGACTTCCAGCGCA

Name	Sequence	PAM	Strand
**sgRNA +44**	CAGGTCGGGCACGTCCGCCA	GGG	-

**sgRNA +15**	GTTACGGGTCGACCTCCAGA	AGG	-
**sgRNA +1**	GGGCGTAGGGCTTCAGTTAC	GGG	-
**sgRNA +1T**	CGTAGGGCTTCAGTTAC	GGG	-
**sgRNA -1**	GGGGCGTAGGGCTTCAGTTA	CGG	-
**sgRNA -1T**	GCGTAGGGCTTCAGTTA	CGG	-
**sgRNA -13**	GTAACTGAAGCCCTACGCCC	CGG	+

Supplementary Table 1. Sequence of sgRNA Targeting the GBE1 gene. In this table the C>A Mutation is shown in red.

Silenced PAM

Treatment	n Sequenced	% INDELS	% HR
No Compound	17	70.6	0.0
15µM RS-1	6	83.3	0.0
80µM SCR7	10	90.0	0.0
Combined	9	88.9	0.0

Supplementary Table 2. RS-1 and SCR7 effect on sgRNA +15 with a (168-30) ssODN with a silenced PAM mutation. Comparisons of the RAD-51 stimulatory compound RS-1 and the DNA-Ligase IV inhibitory compound SCR7 or their combination when using sgRNA+15 with a ssODN with a silence PAM mutation. No HR positive colonies were obtained. No differences were observed between treatment groups using a chi-square test (P<0.05).

Supplementary Dataset 1. NGS reveals proper correction of GBED mutation a well as INDELS at the target site. CRIS.py analysis of NGS results indicating the heterozygous nature of the GBED carrier (yellow

box). The column, #1 Reads, indicates that no INDELS were present in the control or cell lines 1-6, indicating proper HDR of the ssODN. However, cell lines 7-9 contained INDELS (tan boxes) outside the guide target site, with cell line 8 also exhibiting an INDEL disrupting the guide site (light blue box). Ratios in the columns, SNP-test (0.95-1.08) and raw_wt_counter (1.1-1.2), confirm that no mutations or large deletions were present in these cell lines that would limit the ability to detect proper HDR.

Supplementary Dataset 2. Probability of computationally predicted off-target effects for guide RNAs -1, +1 and their truncated versions. List of predicted off target sites and ranking by probability of CRISPR/Cas mediated interaction at the off target loci.

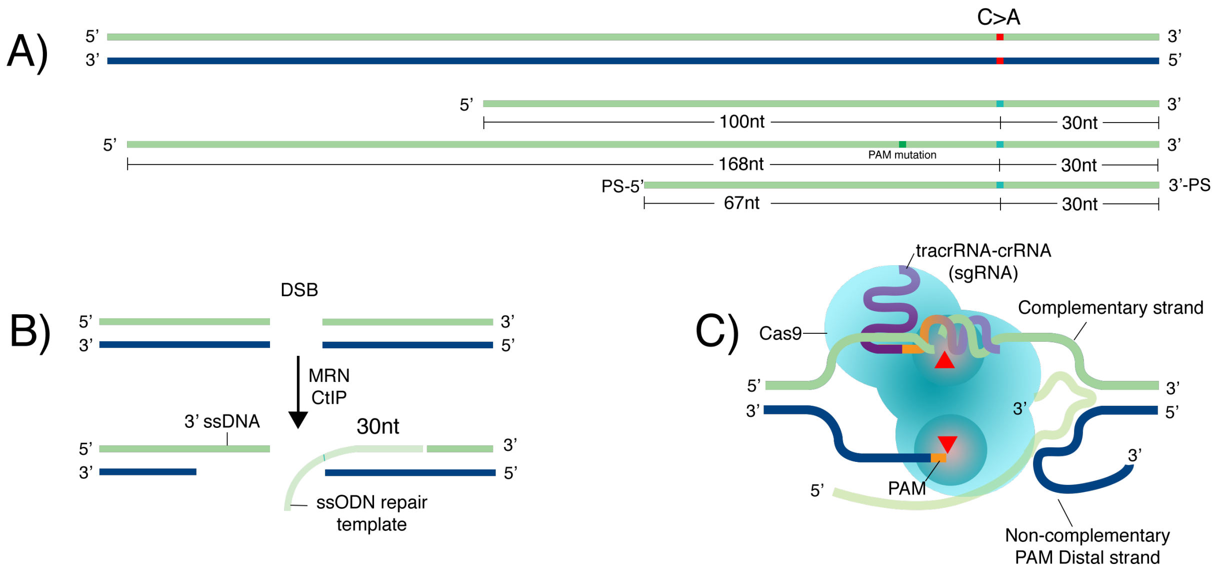

Supplementary Figure 1. Repair template design and theorized repair of ssDNA donor. A) Illustration of the different single-stranded oligodeoxynucleotide (ssODN) repair templates centered at the GBE 102C>A mutation. PS: phosphorothioate modifications. B) First model of ssODN mediated homologous recombination. Here, after the DSB has occurred the carboxy-terminal binding protein interacting protein (CtIP) and the MRN (MRE11–RAD50–NBS1 (Nijmegen breakage syndrome protein 1)) complex create

extensive 5′ to 3′ resection at DSB sites to generate stretches of 3’ single-stranded DNA (ssDNA) ends. The ssODN can then bind to these 3’ ssDNA ends. C) Second model of ssODN repair. Cas9 induces a double stranded DNA break (DSB) after extensive RNA:DNA heteroduplex pairing between the sgRNA and the target sequence. More specifically , Cas9 suffers a conformational change that allows the HNH domain [103] to cleave the gRNA complementary strand (3nt 5’ of the PAM) and the RuvC domain to cleave the non-complementary strand. This second break can be in a variable location because this strand can enter and exit the nuclease domain [70]. After the DSB Cas9-DNA complex remains bound to the cleaved products for ~6h partially releasing the PAM-distal non-target strand before complete dissociation [103, 104]. This partial release allows for the ssODN to bind the PAM-distal non-target strand and binding results in strand migration and extrusion.
